# Supplementary material for: Alterations in Immune Response Profile of Tumor-Draining Lymph Nodes after High-Intensity Focused Ultrasound Ablation of Breast Cancer Patients
Source: Cells. 2021 Nov 29;10(12):3346. doi: 10.3390/cells10123346 (PMC8699337; doi:10.3390/cells10123346)
Supplement: Supplementary file 1 [file cells-10-03346-s001.zip › cells-1358333-SI.pdf]

Supplementary Table S1 Primary Antibodies Used for Immunohistochemical Staining

Antigen	Clone	Isotype	Location of positive staining	Major specificity	Supplier
CD3	PS1	IgG2a	Cell membrane	T lymphocytes	Maxim
CD4	1F6	IgG1	Cell membrane	Helper/inducer T lymphocytes	Novocastra
CD8	C8/144B	IgG1/k	Cell membrane, cytoplasm	Cytotoxic/suppressor T lymphocytes	Maxim
CD20	L26	IgG2a	Cell membrane	B lymphocytes	Maxim
CD57	HK1	IgM/k	Cell membrane	Natural killer cells	Maxim
Fas ligand	5D1	IgM	Cytoplasm	Cytotoxic T lymphocytes, natural killer cells	Maxim
Granzyme	GZB01	IgG2a	Cytoplasm	Cytotoxic T lymphocytes, natural killer cells	Maxim
Perforin	5B10	IgG1	Cytoplasm	Cytotoxic T lymphocytes, natural killer cells	Novocastra

Supplementary Table S2 Clinical and Pathological Characteristics of the Breast Cancer Patients

	Control Group	HIFU Group	*P* Value
No. of patients	25	23	
Age (years)	45.5 ± 1.2	46.5 ± 1.7	.87
Range	29 - 70	23 - 60	
Lymph node tumor involvement			.24
N0 (Tumor-free)	13 (52%)	12 (52.2%)	
N1 (1-3)	6 (24%)	5 (21.7%)	
N2 (4-9)	6 (24%)	6 (26.1%)	
TNM stage			.51
I	2 (8%)	2 (8.7%)	
II	22 (88%)	21 (91.3%)	
III	1 (4%)	0 (0%)	
Histological diagnosis			.46
Infiltrating duct	19 (76%)	21 (91.3%)	
Lobular carcinoma in situ	2 (8%)	2 (8.7%)	
Medullary	2 (8%)	0 (0%)	
Mucinous	2 (8%)	0 (0%)	
Tumor size (cm)	3.5 ± 0.23	3.1 ± 0.79	.76
Range	1.8 - 5.6	2.0 - 4.7	
